# Emergence of Melioidosis in Indonesia

**DOI:** 10.4269/ajtmh.15-0292

**Published:** 2015-12-09

**Authors:** Patricia M. Tauran, Nurhayana Sennang, Benny Rusli, W. Joost Wiersinga, David Dance, Mansyur Arif, Direk Limmathurotsakul

**Affiliations:** Department of Clinical Pathology, Faculty of Medicine, Hasanuddin University/Dr. Wahidin Sudirohusodo Hospital, South Sulawesi, Indonesia; Center of Tropical Medicine and Travel Medicine, Division of Infectious Diseases, Department of Medicine, Academic Medical Center, University of Amsterdam, Amsterdam, The Netherlands; Lao-Oxford-Mahosot Hospital–Wellcome Trust Research Unit, Vientiane, Lao People's Democratic Republic; Centre for Tropical Medicine and Global Health, University of Oxford, Oxford, United Kingdom; Department of Tropical Hygiene, Faculty of Tropical Medicine, Mahidol University, Bangkok, Thailand; Mahidol-Oxford Tropical Medicine Research Unit, Faculty of Tropical Medicine, Mahidol University, Bangkok, Thailand

## Abstract

Melioidosis is known to be highly endemic in parts of southeast Asia and northern Australia; however, cases are rarely reported in Indonesia. Here we report three cases of melioidosis in Makassar, South Sulawesi, Indonesia occurring between 2013 and 2014. Two patients died and the other was lost to follow-up. *Burkholderia pseudomallei* isolates from all three cases were identified by the VITEK2 Compact installed in the hospital in 2012. None of the three patients reported received antimicrobials recommended for melioidosis because of the delayed recognition of the organism. We reviewed the literature and found only seven reports of melioidosis in Indonesia. Five were reported before 1960. We suggest that melioidosis is endemic throughout Indonesia but currently under-recognized. Training on how to identify *B. pseudomallei* accurately and safely in all available microbiological facilities should be provided, and consideration should be given to making melioidosis a notifiable disease in Indonesia.

## Introduction

Melioidosis is a frequently fatal community-acquired infectious disease caused by the environmental gram-negative bacillus *Burkholderia pseudomallei*. The disease lacks a specific clinical presentation, and the current diagnostic gold standard is culture. However, *B. pseudomallei* can be misidentified as a culture contaminant or as another species, especially by laboratory staff unfamiliar with this organism.^1^ The crude case fatality rate for melioidosis ranges from 14% to 40%, and could be as high as 80% if effective antimicrobial drugs are not given.2 Melioidosis is known to be highly endemic in parts of southeast Asia and northern Australia.3 Both human and animal melioidosis cases exported from Indonesia have been frequently observed in many countries4–8; however, indigenous cases are rarely reported in Indonesia. Here, we report three cases of culture-confirmed melioidosis presenting at Wahidin Hospital, South Sulawesi, Indonesia, between 2013 and 2014.

## Case 1

**Figure 1.**
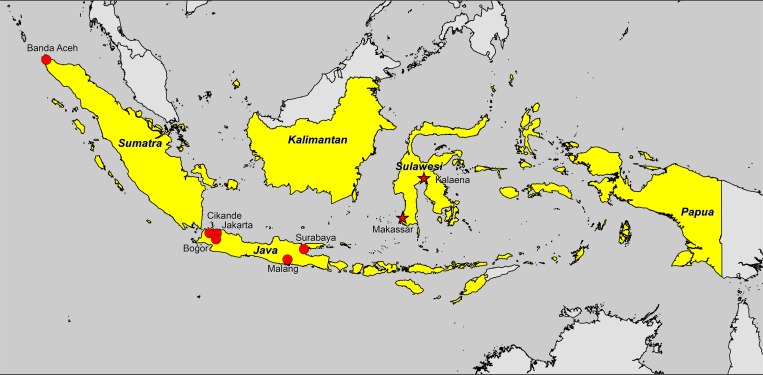
Map of reported indigenous melioidosis cases in Indonesia. Locations of the previous case reports are indicated by red circles (Banda Aceh, Jakarta, Cikande, Bogor, Malang, and Surabaya). Locations of the current case reports are indicated by red stars (Makassar and Kalaena).

A 41-year-old Indonesian male was referred from I La Galigo Hospital, East Luwu Regency, to Wahidin Hospital in August 2012 with a 5-day history of fever, chill, shortness of breath, headache, and confusion. The patient was an excavator operator working in Tambak Yoso village, Kalaena District, East Luwu Regency (Figure 1

), and had no history of underlying diseases.

On admission, the physical examination revealed fever (39.8°C), icteric sclera, hepatomegaly, abdominal tenderness, and calf tenderness. The blood test results showed leukopenia (2,700/μL), thrombocytopenia (37,000/μL), hyperglycemia (350 mg/dL), hyperbilirubinemia (total bilirubin 4.4 mg/dL and direct bilirubin 3.9 mg/dL) and hypercreatinemia (4.5 mg/dL). Rapid diagnostic tests for leptospirosis, dengue infection, and malaria were all negative. He was presumptively diagnosed as having Weil's disease and type 2 diabetes mellitus. Intravenous ceftriaxone and subcutaneous insulin were initiated. He died the following day because of septic shock. His blood culture became positive for *B. pseudomallei* 5 days after admission.

## Case 2

A 45-year-old Indonesian female was referred from Daya Regional Hospital, Makassar, to Wahidin Hospital in July 2013 with a 3-month history of swelling on the right side of the neck. During the past month, the mass became bigger, ruptured, and produced thick, white pus. She also had fever, abdominal pain, vomiting, diarrhea and poor appetite, and had lost 5 kg during the previous month. The patient was a housewife living in Makassar and had a 3-year history of diabetes mellitus and hypertension.

On admission, she was febrile (38.1°C) with a discharging neck abscess that was swabbed for culture. The blood test results showed hyperglycemia (310 mg/dL), while the complete blood count, renal function tests, and liver function tests were normal. Chest radiography showed cardiomegaly with pulmonary edema and signs of pulmonary hypertension. Her sputum examination was negative for acid-fast bacilli. She was diagnosed as suspected tuberculous lymphadenitis and type 2 diabetes mellitus. Intravenous ceftriaxone, subcutaneous insulin, and oral meloxicam and metronidazole were initiated. On the 4th day, she developed dyspnea, and chronic heart failure due to coronary artery disease was diagnosed by a cardiologist. On the 6th day, she died and her cause of death was recorded as cardiogenic shock. Pus culture was reported positive for *B. pseudomallei* 7 days after admission.

## Case 3

A 26-year-old Indonesian male presented at Ear Nose Throat (ENT) Clinic, Wahidin Hospital, Makassar, in February 2014 with a 10-day history of an abscess behind his left ear that was not improving despite treatment. Ten days before presenting at the Wahidin hospital, he had noticed a painful, erythematous, and immobile marble-sized lump behind his ear. The mass had been incised and drained at a private hospital in Makassar and he was given an unknown medication. He was an employee of a State-owned enterprise and had no history of underlying diseases.

At the ENT clinic, he was afebrile (36.6°C). Physical examination revealed that his hearing was normal and his tympanic membrane was intact. The incised wound was painless. The wound was dressed and oral ciprofloxacin was prescribed. After 8 days, he came back to the ENT clinic complaining of a persistent discharge. A further incision and drainage was performed, and the pus was sent for bacterial culture, which was reported as positive for *B. pseudomallei* 3 days later. However, the patient was lost to follow-up.

*Burkholderia pseudomallei* isolates from all the three cases were identified by the VITEK2 Compact (bioMérieux, Marcy l'Etoile, France), which was installed at the microbiology laboratory of Wahidin Hospital, which is South Sulawesi's main referral hospital, in December 2012. *Burkholderia pseudomallei* had never previously been identified in Wahidin Hospital. The isolates were not stored, and were therefore not available for further confirmatory tests.^1^ None of the isolates were tested for antimicrobial susceptibility because this was not included in the laboratory's standard operating procedures (SOPs) for this organism.

We searched PubMed for indigenous cases of melioidosis reported in Indonesia between January 1, 1921 and December 31, 2014, using the MeSH terms “melioidosis” or “pseudomallei.” We also searched bibliographies from selected studies for secondary references (Table 1, Figure 1). Of seven studies identified, five were before 1960 and four were in Dutch.^9^–^13^ Those cases were from Jakarta, Cikande, Bogor, and Surabaya on Java island.

The most recent reports concern four tsunami survivors in Banda Aceh, Sumatra, in 2004,^14^ and 51 culture-confirmed melioidosis patients in Malang, Java, from 2011 to 2013.^15^

This is the first report of indigenous melioidosis cases in Sulawesi. Previous reports of indigenous melioidosis from Indonesia were from Sumatra and Java (Table 1, Figure 1). Considering that melioidosis is endemic in east Malaysia and Papua New Guinea, it is likely that indigenous melioidosis cases also occur unrecognized and unreported in the contiguous parts of Indonesia, Kalimantan and Papua, respectively.3 Our finding strongly supports the suggestion that melioidosis is endemic throughout Indonesia but is currently under-recognized.3

It is likely that *B. pseudomallei* had been isolated in Wahidin hospital before 2012 but was misidentified or discarded as a contaminant.^1^ Guidance on how to diagnose melioidosis and identify *B. pseudomallei*, even without the use of automated machines such as VITEK, was recently published.^1^ *Burkholderia pseudomallei* can also cause laboratory-acquired infection, and appropriate safety precautions for suspected isolates is required.^1^ Since the recognition of these three cases, laboratory SOPs for bacterial identification and susceptibility testing and reporting are being revised in the hospital.

None of the three patients reported received antimicrobials recommended for melioidosis2 because of the delayed recognition of the organism. Treatment guidelines for clinicians in the Wahidin hospital are being revised to consider empirical treatment with antimicrobials effective against *B. pseudomallei* in patients presenting with severe sepsis and hyperglycemia.^1^,2

Although *B. pseudomallei* isolates in this study were not available for further confirmation, identification by VITEK2 is generally reliable.^1^ In addition, clinical manifestations of the three cases are consistent with common clinical presentations of melioidosis, including severe sepsis with multiple organ failure (Cases 1 and 2) and localized subcutaneous abscesses that fail to improve after treatment with antimicrobials ineffective against *B. pseudomallei* (Case 3).

The melioidosis cases in Sulawesi reported here are likely to represent the tip of the iceberg in Indonesia. We suggest that training on how to identify *B. pseudomallei* accurately and safely in all available microbiological facilities in Indonesia should be provided. In addition, consideration should be given to making melioidosis a notifiable disease in Indonesia.

## Figures and Tables

**Table 1:** Reported indigenous human cases of melioidosis in Indonesia

Year presented (reference)	Locations	Age (years)/gender, nationality	Clinical characteristics	Diagnostic method (bacterial identification method)	Outcome
1929^9^	Cikande, Java	50/M, Indonesian	Chronic painless nodules in the left thigh with fistula discharging greenish yellow pus	Culture of pus (biochemical, phenotypic tests and virulence in animal model))	Died
1934^10^	Jakarta, Java	38/M, Indonesian	Severe sepsis with pulmonary, splenic, and prostatic abscesses (postmortem)	Culture of pus (biochemical, phenotypic tests and virulence in animal model)	Died
1935^11^	Surabaya, Java	25/F, Indonesian	Abscess in the right gluteal region	Culture of pus (biochemical, phenotypic tests and virulence in animal model)	Fully recovered
1936^12^	Bogor, Java	60/M, Indonesian	Skin lesion with ulcers on right lower leg after trauma	Culture of pus (biochemical and phenotypic tests)	Fully recovered
1937^12^	Jakarta, Java	55/M, Indonesian	Abscess left foot, originated from small trauma while farming	Culture of pus (biochemical and phenotypic tests)	Fully recovered
1950^13^	Surabaya, Java	28/F, European	Pain in the lower abdomen and then high fever	Culture of abscess from the right ovary (biochemical and phenotypic tests)	Fully recovered
2005^14^	Banda Aceh, Sumatra	15/F; 18 months/M; 10/F; 13/F (four tsunami survivors)	Pneumonia	Culture of sputum (API20NE)	Fully recovered (*N* = 1) or reported as improving (*N* = 3)
2011–2013^15^	Malang, Java	51 patients	Unknown	Culture of sputum, blood, pus, and urine (VITEK2)	Unknown
2013 (Case 1)	Luwu Timur, Sulawesi	41/M, Indonesian	High grade fever, chill, headache, and shortness of breath	Culture of blood (VITEK2)	Died
2013 (Case 2)	Makassar, Sulawesi	45/F, Indonesian	Skin ulcer on neck, fever, vomiting, abdominal pain, headache, diarrhea, poor appetite, and weight loss	Culture of pus (VITEK2)	Died
2013 (Case 3)	Makassar, Sulawesi	26/M, Indonesian	Purulent discharge from incised wound behind the left ear lobe, painless and no fever	Culture of pus (VITEK2)	Lost to follow-up

F = female; M = male.

## References

[1] Hoffmaster AR, AuCoin D, Baccam P, Baggett HC, Baird R, Bhengsri S, Blaney DD, Brett PJ, Brooks TJ, Brown KA, Chantratita N, Cheng AC, Dance DA, Decuypere S, Defenbaugh D, Gee JE, Houghton R, Jorakate P, Lertmemongkolchai G, Limmathurotsakul D, Merlin TL, Mukhopadhyay C, Norton R, Peacock SJ, Rolim DB, Simpson AJ, Steinmetz I, Stoddard RA, Stokes MM, Sue D, Tuanyok A, Whistler T, Wuthiekanun V, Walke HT (2015). Melioidosis diagnostic workshop, 2013. Emerg Infect Dis.

[2] Lipsitz R, Garges S, Aurigemma R, Baccam P, Blaney DD, Cheng AC, Currie BJ, Dance D, Gee JE, Larsen J, Limmathurotsakul D, Morrow MG, Norton R, O'Mara E, Peacock SJ, Pesik N, Rogers LP, Schweizer HP, Steinmetz I, Tan G, Tan P, Wiersinga WJ, Wuthiekanun V, Smith TL (2012). Workshop on treatment of and postexposure prophylaxis for *Burkholderia pseudomallei* and *B. mallei* infection, 2010. Emerg Infect Dis.

[3] Currie BJ, Dance DA, Cheng AC (2008). The global distribution of *Burkholderia pseudomallei* and melioidosis: an update. Trans R Soc Trop Med Hyg.

[4] Beeker A, Van de Stadt KD, Bakker K (1999). Melioidosis. Neth J Med.

[5] Dance DA, Smith MD, Aucken HM, Pitt TL (1999). Imported melioidosis in England and Wales. Lancet.

[6] Lee SW, Yi J, Joo SI, Kang YA, Yoon YS, Yim JJ, Yoo CG, Han SK, Shim YS, Kim EC, Kim YW (2005). A case of melioidosis presenting as migrating pulmonary infiltration: the first case in Korea. J Korean Med Sci.

[7] Dance DA, King C, Aucken H, Knott CD, West PG, Pitt TL (1992). An outbreak of melioidosis in imported primates in Britain. Vet Rec.

[8] Ritter JM, Sanchez S, Jones TL, Zaki SR, Drew CP (2013). Neurologic melioidosis in an imported pigtail macaque (*Macaca nemestrina*). Vet Pathol.

[9] de Moor CE, Soekarnen, van der Walle N (1932). Melioidosis op Java. Geneeskd Tijdschr Ned Indie.

[10] Pet MA, Fossen A (1934). Melioidosis der inwendige organen [Melioidosis of internal organs]. Geneeskd Tijdschr Ned Indie.

[11] Bezemer F (1935). Melioidosis op Celebes. Geneeskd Tijdschr Ned Indie.

[12] Sudibyo RMS (1938). Twee gevallen van huidmelioidosis. Geneeskd Tijdschr Ned Indie.

[13] Dunlop SJ (1952). Rapid recovery in a case of melioidosis. Doc Med Geogr Trop.

[14] Athan E, Allworth AM, Engler C, Bastian I, Cheng AC (2005). Melioidosis in tsunami survivors. Emerg Infect Dis.

[15] Irmawanti-Rahayu S, Noorhamdani AS, Santoso S (2014). Resistance pattern of *Burkholderia pseudomallei* from clinical isolates at Dr. Saiful Anwar General Hospital, Malang-Indonesia. J Clin Microbiol Infect Dis.

